# A Potential Role for HUWE1 in Modulating Cisplatin Sensitivity

**DOI:** 10.3390/cells10051262

**Published:** 2021-05-20

**Authors:** Stijn Wenmaekers, Bastiaan J. Viergever, Gunjan Kumar, Onno Kranenburg, Peter C. Black, Mads Daugaard, Richard P. Meijer

**Affiliations:** 1 Laboratory Translational Oncology, University Medical Center Utrecht, 3584CX Utrecht, The Netherlands; s.wenmaekers-3@umcutrecht.nl (S.W.); B.J.Viergever-3@umcutrecht.nl (B.J.V.); o.kranenburg@umcutrecht.nl (O.K.); 2 Department of Oncological Urology, University Medical Center Utrecht, 3584CX Utrecht, The Netherlands; 3 Department of Urologic Sciences, University of British Columbia, Vancouver, BC V5Z 1M9, Canada; gkumar@prostatecentre.com (G.K.); peter.black@ubc.ca (P.C.B.); 4 Vancouver Prostate Centre, Vancouver, BC V6H 3Z6, Canada

**Keywords:** chemotherapy, resistance, biomarkers, translational medicine research, ubiquitination, DNA damage response, apoptosis

## Abstract

Cisplatin is a widely used antineoplastic agent, whose efficacy is limited by primary and acquired therapeutic resistance. Recently, a bladder cancer genome-wide CRISPR/Cas9 knock-out screen correlated cisplatin sensitivity to multiple genetic biomarkers. Among the screen’s top hits was the HECT domain-containing ubiquitin E3 ligase (HUWE1). In this review, HUWE1 is postulated as a therapeutic response modulator, affecting the collision between platinum-DNA adducts and the replication fork, the primary cytotoxic action of platins. HUWE1 can alter the cytotoxic response to platins by targeting essential components of the DNA damage response including BRCA1, p53, and Mcl-1. Deficiency of HUWE1 could lead to enhanced DNA damage repair and a dysfunctional apoptotic apparatus, thereby inducing resistance to platins. Future research on the relationship between HUWE1 and platins could generate new mechanistic insights into therapy resistance. Ultimately, HUWE1 might serve as a clinical biomarker to tailor cancer treatment strategies, thereby improving cancer care and patient outcomes.

## 1. Introduction

Cisplatin is among the most widely used chemotherapeutical drugs since it was clinically introduced in 1978 [1,2]. Despite the implementation of advanced generation cisplatin analogues such as carboplatin and the emerging field of targeted cancer therapies, cisplatin remains a first-line drug for various malignancies including muscle invasive bladder cancer, testicular germ cell tumors, and non-small cell lung cancer [3,4,5].

Platinum-based therapy is believed to exert its main cytotoxic effect by covalently binding DNA purine bases to form intra-and interstrand crosslinks [6,7]. These lesions disrupt the DNA structure and interfere with the transcription and replication machinery. The crucial cytotoxic event induced by platins is the collision of replication forks (RFs) with bulky platinum-DNA adducts during the cell cycle’s S phase [8]. Cells respond to these lesions throughout the cell cycle via sophisticated DNA damage response (DDR) mechanisms. Initially, DNA damage repair pathways become active in an attempt to maintain genomic integrity. However, prolonged RF stalling at irreparable lesions leads to double stranded DNA breaks (DSBs), genomic instability, and eventually apoptosis [9]. Interstrand crosslinks (ICLs) comprise less than 5% of all platinum-DNA adducts and are the most cytotoxic lesions as they prevent DNA strand separation during replication, thereby inducing a powerful apoptotic stimulus [9,10]. In addition to DNA crosslinking, platins mediate cytotoxicity via other mechanisms including the elevation of intracellular reactive oxygen species [11].

Cellular sensitivity to cancer therapy depends on a dynamic variety of molecular tumor signatures [12]. This translates to intrinsic and acquired resistance to platins. Most cancer cells are initially susceptible to platins, but over time, sensitivity often decreases. Underlying mechanisms of resistance are complex and include reduction of cellular drug availability via efflux pumps and/or enhancing the removal and repair of platinum-DNA adducts. Ultimately, mechanisms of resistance to platins work synergistically to prevent, tolerate, and resolve DNA damage, thereby evading apoptosis and resulting in cancer cell immortalization [13,14].

Integrating incremental understanding of tumor heterogeneity with the exponential increase in available cancer therapies, the urge to select patients based on individual tumor characteristics prior to treatment has become increasingly relevant. Challenges in developing tailored treatment strategies to optimize outcomes are not limited to platins, but apply to all cancer drugs [15]. To improve cancer care, identification of reliable biomarkers that assess and predict a patient’s individual response to treatment is of outmost importance.

Introduction of the CRISPR/Cas9 genome editing technique has revolutionized genetic screening for biomarker identification purposes [16]. Effective CRISPR/Cas9 gene knock-out (KO) screening enables researchers to functionally study the relationship between genes and specific cell phenotypes. Applying this technique on a genome wide scale provides a highly effective method for unbiased identification of genes related to cancer therapy resistance [17]. Recently, a bladder cancer genome-wide CRISPR/Cas9 KO screen correlated several genes to cisplatin sensitivity [18]. HUWE1 KO was among the screen’s top hits and strongly correlated to cisplatin resistance.

HUWE1 is a ubiquitin (Ub) E3 ligase that functions as a terminating enzyme in the process of protein ubiquitination. Following the consecutive actions of Ub-activating (E1) and -conjugating (E2) enzymes, HUWE1 post-translationally modifies other proteins by adding different types of Ub chains. A well-known function of HUWE1 is that it targets other proteins for degradation in the Ub-proteasome system (UPS) via K48-linked poly-Ub chains [19]. In contrast to being a facilitator of protein degradation, HUWE1 regulates processes such as protein activation and cellular signal transduction via K63-linked polyubiquination [19,20]. HUWE1-mediated monoubiquitination and less well understood K6-linked polyubiquitination further indicate its multifaceted cellular regulatory effects [21,22,23,24]. Deubiquitinating enzymes on the other hand counteract the post-translational actions of HUWE1. In addition to the catalytic HECT domain, HUWE1 contains a Ub-associated (UBA) domain, a Ub-binding motif (UBM1) domain, and a Bcl-2 homology region 3 (BH3) domain [25,26]. While the functions of the UBM1- and UBA domains are still obscure, evidence indicates that the BH3 domain allows HUWE1 to specifically interact with the induced myeloid leukemia cell differentiation protein (Mcl-1), an anti-apoptotic member of the B-cell lymphoma 2 (Bcl-2) family [27]. This interaction implies a modulatory role for HUWE1 in apoptosis. Many other HUWE1 targets with roles in the DDR have already been identified [28].

HUWE1-mediated ubiquitination is a highly coordinated process and deregulation has been linked to tumorigenesis as well as tumor suppression [25,26]. Given its modulatory role on mediators such as Mcl-1, the breast cancer type 1 susceptibility protein (BRCA1) and p53, HUWE1 is linked to DNA damage repair pathways and apoptosis [27,29,30]. These processes are not only hallmarks of cancer, but also influence cellular sensitivity to platins and other genotoxins [31,32]. In this review, HUWE1 is postulated as a modulator of platinum-based therapy sensitivity by promoting collision between RFs and platinum-DNA adducts, the primary cytotoxic action of platins. By addressing this topic, we aim to provide a mechanistic framework in support of our recent observations on HUWE1 in bladder cancer [18]. Cellular deficiency of HUWE1 might drive resistance to platins via enhanced DNA damage repair and evasion of apoptosis, thereby sparking HUWE1′s biomarker potential. Ultimately, HUWE1 could become valuable in guiding therapy decision-making, overcoming resistance to platins, and as a future target of novel treatment strategies. 

## 2. HUWE1 Interferes with DNA Damage Repair and Tolerance

DDR pathways comprise of multiple levels and generally follow a consecutive order of events to maintain genomic stability. Firstly, proteins from the phosphoinositide 3-kinase (PI3K) protein family sense replicative stress or DNA damage. Subsequently, the signal is cascaded and amplified to finally activate DDR effector proteins. These effectors have pleiotropic cellular effects that primarily include cell cycle modulation, DNA damage repair or tolerance, and ultimately apoptosis to protect from mutagenesis. Essential P13K sensors are the ataxia-telangiectasia mutated (ATM) and the ataxia-telangiectasia and Rad3-related (ATR) proteins which are at the apex of the prominent ATR-checkpoint kinase 1 (Chk1) and ATM-checkpoint kinase 2 (Chk2) DDR pathways [33].

Several interference possibilities of HUWE1 with DDR pathways and the primary cytotoxic actions of platins are known. These affect the relationship between RFs and platinum-DNA adducts, further subdivided into prevention of RF collision (Section 2.1), resolution of replicative stress at stalled RFs (Section 2.2), and repair of DNA damage after prolonged RF stalling (Section 2.3). Additionally, HUWE1 interferes with the intrinsic apoptotic pathway that is the DDR executioner of platinum-based therapy’s cytotoxicity (Section 3). 

### 2.1. Interplay between HUWE1 and Mechanisms That Prevent Replication Fork Collision with Platinum-DNA Adducts

Prior to encountering RFs, platinum-DNA adducts are primarily processed via the nucleotide excision repair (NER) system. Initial NER sensing of such lesions occurs via distinct mechanisms, known as the transcription-coupled or global genomic pathway. After sensing, NER pathways converge and endonucleases excise the lesion. New DNA is subsequently synthesized at the freshly formed single-stranded DNA (ssDNA) gap to restore the normal DNA helical structure [34]. The NER system is highly effective in the processing of intrastrand crosslinks [35]. However, ICLs can also be resolved by two consecutive rounds of NER and the use of translesion synthesis (TLS) polymerases. Factors of the NER system are not merely involved in lesion processing prior to encounter with the RF, but also facilitate more complex replication-coupled ICL repair [36,37].

**Figure 1:**
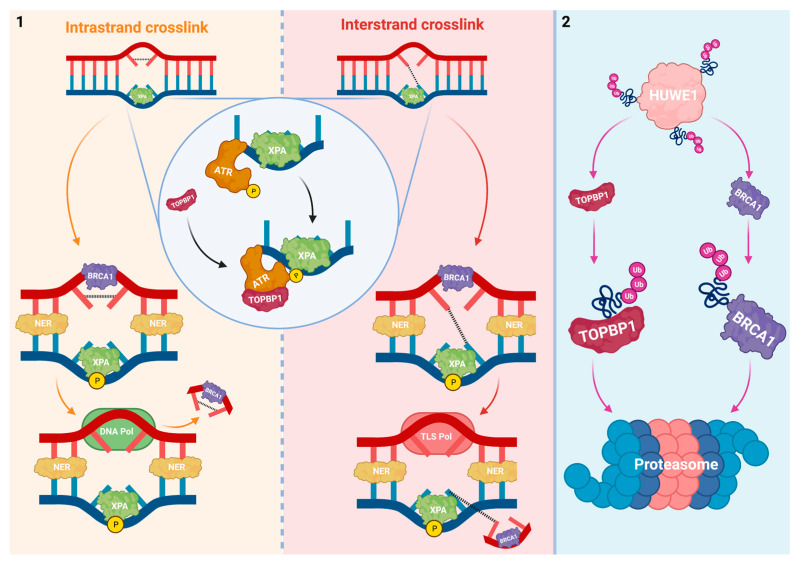
HUWE1 interferes with mediators of NER. (**1**) To phosphorylate XPA and activate NER at intra- and interstrand crosslinks, ATR is first activated by TOPBP1. After NER activation, the lesion is excised by NER factors and new DNA is synthesized at the ssDNA gap. ICLs are resolved via two consecutive rounds of NER and with the use of TLS polymerases. (**2**) HUWE1 negatively regulates TOPBP1 via polyubiquitination. The breast cancer type 1 susceptibility protein (BRCA1) * promotes NER and is negatively regulated by HUWE1 via polyubiquitination. HUWE1 depletion could hyperactivate NER and the processing of platinum-DNA adducts via overexpression of its downstream targets. * Discussed in Section 2.3.

The Xeroderma pigmentosum complementation group A (XPA) protein has a central role within NER and is considered the rate-limiting factor. To promote NER, XPA is phosphorylated by ATR [38]. However, ATR needs to be activated itself first by the topoisomerase 2-binding protein 1 (TOPBP1) to exert its kinase activity on XPA [39,40]. HUWE1 is a regulator of TOPBP1 and therefore it potentially modulates NER-mediated processing of platinum-DNA adducts (Figure 1).

TOPBP1 is ubiquitinated for proteasomal degradation by HUWE1 in human colon carcinoma (Ls174T) cells [41]. Ectopic HUWE1 expression led to substantial ubiquitination of soluble TOPBP1 in these cells. Ubiquitination was on the other hand virtually absent in HUWE1 deficient conditions. In addition, TOPBP1 was protected from HUWE1′s negative regulation by complex formation with the Myc-interacting zinc finger protein 1 (Miz1) [41].

Interestingly, HUWE1-mediated turnover of TOPBP1 seems related to the cell’s physiological circumstance. Whereas TOPBP1 formed complexes with Miz1 in unstressed cells, ultra-violet (UV)-irradiation-induced cellular stress led to complex dissociation. The subsequent HUWE1-mediated turnover of unbound TOPBP1 was important to terminate ATR-dependent signaling in these stressed cells. Other E3 Ub ligases have previously been implicated in TOPBP1 degradation. However, depletion of these alternative E3 Ub ligases had no effect on TOPBP1 levels [41]. This proposes HUWE1 as a predominant regulator of TOPBP1 and ATR activity. Enhancement of TOPBP1 due to HUWE1 deficiency could lead to NER hyperactivation via its downstream effects on XPA. Initiation of a cytotoxic response could be prevented via excessive NER-mediated processing of platinum-DNA adducts.

The direct relationship between HUWE1, TOPBP1, and sensitivity to platins remains to be established. However, TOPBP1 expression levels have been correlated to cisplatin resistance and poor patient outcomes [42,43]. Others have already indicated the potential of TOPBP1 as a future cancer drug target [44]. One should note, however, that the interplay between HUWE1 and other TOPBP1 E3 ligases could vary in spatiotemporal dimensions. To illustrate this, normal TOPBP1 levels may be maintained by compensatory upregulation of other E3 Ub ligases after loss of HUWE1. Existence of an alternative pathway for ATR activation, mediated by the Ewing’s tumor-associated antigen 1, further strengthens the concept that the significance of HUWE1-mediated effects on NER may be context-specific [45].

The NER system has been extensively linked to platinum-based therapy sensitivity in various malignancies [35,46,47,48,49]. Moreover, the success of cisplatin in curing metastatic testicular germ cell tumors has been attributed to impaired NER-mediated DNA damage repair [49]. Next to DNA endonucleases, that are believed to be the most potent NER modulators of sensitivity to platins, XPA also influences the drug response [50,51]. A recent report correlated XPA expression levels in germ cell tumors to an aberrant cisplatin response and identified it as an independent prognostic biomarker for poor patient outcomes [52]. While others have clearly indicated the significance of XPA phosphorylation on its stability, increased XPA levels in cisplatin resistant germ cell tumor cells could not be linked to its phosphorylation status [38,52]. Targeting XPA with specific inhibitors has been studied pre-clinically. Especially compounds that prevent the interaction between XPA and NER endonucleases should be considered as promising future sensitizers of platinum-based therapy [53,54].

Apart from its discussed role on NER-mediated processing of DNA lesion prior to RF encounter, HUWE1′s effects on TOPBP1 can alter other levels of the cytotoxic response to platins. TOPBP1-mediated activation of ATR namely lies at the apex of the prominent ATR-Chk1 pathway that becomes active when RFs collide with platinum-DNA adducts (Section 2.2). Furthermore, TOPBP1 has been reported to directly promote homologous recombination (HR)-mediated DSB repair (Section 2.3) [55]. HUWE1-induced TOPBP1 turnover thus does not merely influence NER but also affects a subset of other important DDR systems that modulate sensitivity to platinum-based therapy.

Loss of HUWE1 confers risk of resistance to platins via TOPBP1 enhancement and hyperactivation of NER-mediated DNA repair. Genotoxic platinum-DNA adducts that form the basis for a further cytotoxic response can be nullified in such manner. This makes HUWE1 a potential biomarker to assess the responsiveness to platins based on mechanisms that prevent RFs from colliding with platinum-DNA adducts. Stimulation of HUWE1 might promote TOPBP1 degradation to blunt DDR signaling and potentiate platinum-based compounds. 

### 2.2. Interplay between HUWE1 and Pathways That Are Activated upon Replication Fork Collision with Platinum-DNA Adducts

At the encounter between RFs and platinum-DNA adducts, intrastrand crosslinks can be bypassed by low-fidelity TLS polymerases but ICLs induce an absolute replicative block [10,56]. A complex and coordinated interplay of multiple DNA damage repair and tolerance mechanisms including NER, TLS, and HR facilitates replication-coupled ICL repair to overcome these blocks [36,37]. The ATR-Chk1 signaling pathway acts as an important DDR orchestrator in this process. HUWE1 interacts with the ATR-Chk1 response, TLS, and HR. Thereby, it potentially affects replication-coupled ICL repair at the stalled RF. Considering its predominant involvement in DSB repair, the relationship between HUWE1 and HR factors is elaborated upon later (Section 2.3). Stabilization and restart of the RF independent of genomic DSBs is however also linked to HR [57].

**Figure 2:**
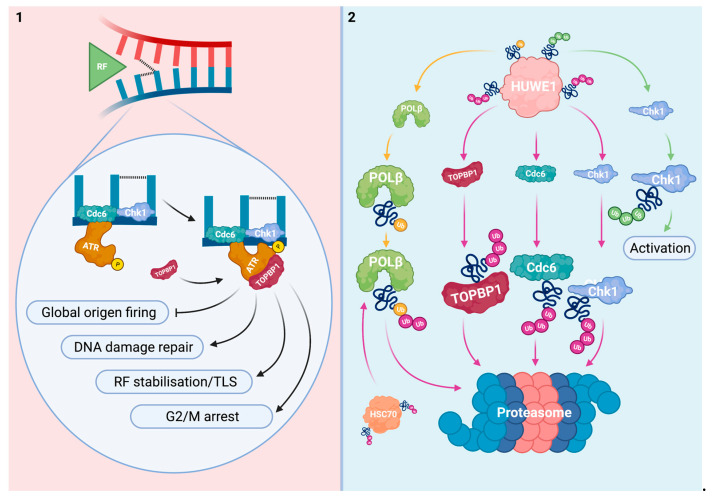
HUWE1 regulates mediators of the ATR-Chk1 signaling pathway that is activated at stalled RFs. (**1**) Upon RF stalling at intra- or interstrand crosslinks, ATR is recruited to the lesion and activated by TOPBP1. Cdc6 functions as a chromatin receptor for ATR. Chk1 is phosphorylated by ATR and triggers a response cascade to mitigate replication stress. This includes modulation of the cell cycle, DNA damage repair, inhibition of global origin firing and RF stabilization/remodeling. Pol β is responsible for TLS at stalled RFs. (**2**) HUWE1 negatively regulates Cdc6, TOPBP1 and Chk1 via polyubiquitination. Alternatively, Chk1 is activated to lesser extend by HUWE1-mediated polyubiquitination. Pol β is monoubiquitinated by HUWE1 prior to Hsc70-interacting protein-mediated polyubiquitination for proteasomal degradation. HUWE1 depletion could hyperactivate the ATR-Chk1 axis via enhancement of its targets.

Stalling of RFs at platinum-DNA adducts leads to excessive unwinding of the double DNA helix and exposure of fragile ssDNA due to persisting DNA helicase activity. These stretches of ssDNA are potent activators of the ATR-Chk1 pathway. Firstly, ATR is recruited to the ssDNA alongside other mediators. Subsequently, TOPBP1 activates ATR and facilitates the signaling relay to Chk1, ATR’s principal target and DDR effector [39,58]. In addition to mediating TOPBP1 degradation, HUWE1 modulates other levels of the ATR-Chk1 response at stalled RFs. With respect to these processes, HUWE1 directly regulates cell division cycle 6 (Cdc6), Chk1, and DNA polymerase beta (Pol β) (Figure 2). 

Cdc6 is best known for coordinating the assembly of pre-replication complexes (preRCs) during G1 phase of the cell cycle [59]. Cellular Cdc6 levels are tightly controlled in rapidly proliferating cells by the Cdh1-activated form of the anaphase promoting complex (APC^Cdh1^), an E3 Ub ligase. In response to DNA damage, Cdc6 degradation is promoted in a p53-dependent manner to prevent DNA replication and protect cells from mutagenesis [60]. Effects of Cdc6 are not limited to the assembly of preRCs in the G1 phase of the cell cycle. Chromatin-bound Cdc6 namely serves as an ATR receptor during S phase. This facilitates ATR retention at DNA lesions and strengthens the ATR-Chk1 axis [61].

In response to genotoxic stress, Cdc6 is ubiquitinated by HUWE1 for proteasomal degradation [62,63]. HUWE1 polyubiquitinates Cdc6 regardless of the cell cycle phase, p53 status or APC^Cdh1^ activity upon UV-irradiation or methyl methane-sulfonate treatment in cervical cancer (HeLa) cells. Interestingly, Cdc6 degradation was impaired by HUWE1 knock-down (KD) in these cells [62]. A similar reciprocal relationship between HUWE1 and Cdc6 was observed in normal fibroblasts (NHF1) upon endogenous DNA damage [63]. Cellular deficiency of HUWE1 thus potentially consolidates the ATR-Chk1 response via upregulation of Cdc6 and subsequent ATR retention at regions of DNA damage. 

Chk1′s principal function is activation of the DNA damage checkpoint [64]. This slows down cell cycle progression during S phase and halts stressed cells at the G2/M transition enabling them to activate DNA damage repair and tolerance mechanisms. Additionally, Chk1 promotes the stabilization and remodeling of stalled RFs to facilitate processes such as TLS and mitigate replication stress. Another important function of Chk1 is the global inhibition of DNA replication origin firing. This prevents replicative problems to arise elsewhere in the genome, thereby protecting cells from replication catastrophe. In contrast, Chk1 promotes the firing of dormant origins in the vicinity of the stalled RF to complete DNA replication [33]. 

HUWE1 ubiquitinates Chk1, as do other E3 Ub ligases including Cullin-4A (Cul4A) [19,65,66]. The putative modulatory role of HUWE1 on Chk1 was first reported in human embryonic kidney (HEK293T) cells [66]. Recently, Chk1 has been further established as a HUWE1 target in osteosarcoma (U2OS) and HeLa cells. Here, HUWE1 predominantly polyubiquitinated active Chk1 with K48-linked chains for proteasomal degradation. Observation of alternative K6- and K63-linked polyubiquitination however stresses the multifactorial regulatory role of the HUWE1/Chk1-complex [19].

Whereas prolonged replication stress normally destabilizes Chk1, HUWE1 KD significantly protected Chk1 from destabilization upon hydroxyurea or camptothecin exposure in HeLa cells. The HUWE1 KD-induced rescue effect on Chk1 was significantly stronger when compared to Cul4A KD [19,65]. These findings postulate HUWE1 as a predominant regulator of Chk1 upon specific genotoxic stimuli. Combined, the relationship between HUWE1 and Chk1 strengthens the concept that HUWE1 deficiency can hyperactivate ATR-Chk1 signaling to mitigate replication stress.

Pol β is a family X DNA polymerase that is best known for its function in short gap DNA synthesis during base excision repair (BER) [67,68]. Additionally, Pol β serves a role in TLS which is promoted by ATR-Chk1 signaling [69]. Effective bypassing of platinum-DNA adducts by Pol β has been demonstrated previously [70]. 

HUWE1-mediated monoubiquitination of Pol β negatively influenced its protein stability in HeLa cells. After priming with mono-Ub on N-terminal residues, Pol β is polyubiquitinated for proteasomal degradation by the Hsc70-interacting protein, another E3 Ub ligase. HUWE1 KD led to Pol β accumulation and enhanced BER-mediated DNA damage repair [21]. Next to BER, loss of HUWE1 function potentially promotes TLS at stalled RFs via upregulation of Pol β, thereby tolerating DNA damage as a consequence of platinum-DNA adducts. Proteasomal degradation of Pol β is however also mediated by C-terminal ubiquitination independent of HUWE1 and the Hsc70-interacting protein in LN428 glioblastoma cells, thereby further indicating context-specific relevance of HUWE1 on protein turnover [71].

Cdc6, Chk1, and Pol β have all been implicated as modulators of sensitivity to platinum-based therapy. However, the direct relationship between HUWE1, these targets, and the responsiveness to platins remains to be established. 

The exact role of Pol β on modulating sensitivity to platins is controversial. Reduction of Pol β levels has been reported to sensitize multiple cell types to cisplatin treatment [72,73]. These observations are in line with Pol β being a TLS polymerase that promotes DNA damage tolerance. Contrastingly, Pol β is reported to interfere with other DDR mechanisms in a manner that impairs the processing of ICLs [74,75]. By this, platinum-DNA adducts remain unresolved to trigger a cytotoxic response when Pol β is expressed. In addition to inconsistent observations regarding Pol β and sensitivity to platins, involvement of many other low-fidelity polymerases in TLS further intricate the interpretation and significance of HUWE1-mediated effects on TLS [76]. 

Interestingly, it was found that Cdc6 functions as a biomarker for cisplatin resistance in bladder cancer models. Reduction of Cdc6 levels resulted in a blunted ATR-Chk1 response and successfully re-sensitized the investigated models to cisplatin treatment [77,78]. Although the correlation with HUWE1 was not yet further investigated, this highlights a potential role for HUWE1 on cisplatin sensitization via Cdc6 downregulation. 

Hyperactivation of ATR-Chk1 signaling is known to negatively impact the cellular sensitivity to various cancer drugs including platins. For this reason, targeting mediators of the ATR-Chk1 axis with specific inhibitors is a rational treatment strategy in oncology. Combining ATR-Chk1 inhibitors with platins has provided synergistic strategies. Especially p53 deficient tumors seem to benefit from ATR-Chk1 inhibition since these rely more heavily on the cell cycle’s G2/M checkpoint for DNA damage repair [79]. The observed K6- and K63-linked polyubiquitination of Chk1 highlights the complex biological role of HUWE1 that is not limited to proteasomal degradation [19]. Polyubiquitination of Chk1 with K63-linked chains results in chromatin association and protein activation. However, this alternative type of polyubiquitination is not HUWE1′s principal effect on Chk1 and CRL4^Cdt2^, another E3 Ub ligase, is involved in this process [80]. This drives the concept that HUWE1 predominantly mediates Chk1 degradation which promotes HUWE1 as an ATR-Chk1 inhibitor. 

Loss of HUWE1 can hyperactivate ATR-Chk1 signaling and promote TLS. These effects may subsequently decrease the cellular responsiveness to platins via mitigation of replication stress. Combined, these data support HUWE1 as a potential biomarker for therapy response assessment regarding mechanisms that become active at stalled RFs. Stimulation of HUWE1 could inhibit the ATR-Chk1 axis at multiple levels and thereby potentiate the cytotoxicity of platinum-based compounds.

### 2.3. Interplay between HUWE1 and Mechanisms Induced by DNA Damage due to Replication Fork Collision with Platinum-DNA Adducts

Prolonged RF stalling at platinum-DNA adducts that cannot be bypassed leads to DSB formation. Multiple etiologies including replication-coupled ICL repair are responsible for DSBs to originate at these sites [36]. DSB repair is mediated by HR and non-homologous end joining (NHEJ). During the cell cycle’s S and G2 phase, HR is the predominant system responsible for DSB repair. This process is more precise and error-proof when compared to NHEJ since it uses a nascent sister chromatid as template [81].

**Figure 3:**
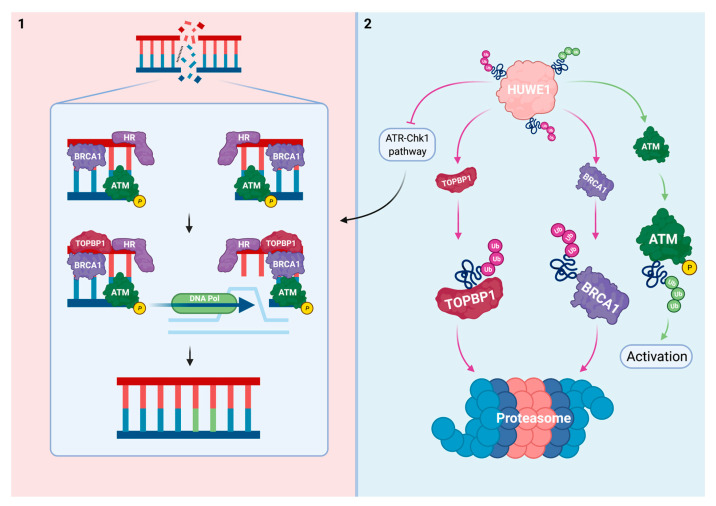
HUWE1 regulates mediators of DSB repair. (**1**) During the S and G2 phase, HR factors including ATM and BRCA1 are recruited to the DSB. ATM is phosphorylated to become active and stimulates BRCA1. HR uses an adjacent sister chromatid to resolve a DSB. (**2**) HUWE1 negatively regulates TOPBP1 and BRCA1 via polyubiquitination. HUWE1 positively regulates ATM phosphorylation. In addition to interfering with the ATM-Chk2 axis for DSB repair, HUWE1 inhibits the ATR-Chk1 response that promotes DSB repair. Loss of HUWE1 could lead to hyperactivation of HR-mediated DSB repair. Impairment of ATM activity in HUWE1 deficient conditions can be compensated for by ATR-Chk1 overactivation.

Although the ATM-Chk2 pathway is primarily triggered in response to DSBs, ATR-Chk1 signaling also promotes the repair of DSBs [33]. HUWE1 interferes with the ATM-Chk2 axis at multiple levels. In regard to these processes, HUWE1 regulates ATM, which is positioned centrally in both DSB repair pathways. Additionally, HUWE1 regulates BRCA1, an ATM substrate with a more distinct role in HR (Figure 3). 

ATM signaling in response to genomic DSBs prevents premature mitotic entry by modulating the cell cycle and promoting DSB repair. An important ATM substrate is the tumor suppressor p53. Contrastingly to ATM’s role in promoting survival and allowing cells to repair DNA damage, ATM-mediated p53 activation is essential to trigger apoptosis in case of persisting replication stress or severe DNA damage (Section 3) [33].

Activation of ATM is mediated by HUWE1 in B-cells and mouse embryonic fibroblasts (MEFs). Upon doxorubicin or y-irradiation treatment, significantly lower levels of phosphorylated ATM accumulated in HUWE1 deficient cells when compared to controls. Immunoprecipitation indicated a specific interaction between HUWE1 and phosphorylated ATM [82]. Loss of HUWE1 function thus impairs ATM signaling. 

BRCA1 is a tumor suppressor protein best known for its role in breast and ovarian cancer [83]. Loss of BRCA1 promotes tumorigenesis and accelerates genomic instability via ineffective HR-mediated DSB repair [84]. Normally, BRCA1 protein levels are tightly controlled by transcriptional and post-transcriptional mechanisms. Once expressed, UPS mediators including the HECT and RLD domain-containing E3 Ub protein ligase 2 antagonize BRCA1 [85]. 

Proteasomal degradation of BRCA1 is also induced by HUWE1-mediated polyubiquitination [29,82,86]. This reciprocal relationship was first observed in MEFs and B-cells [82]. The specific molecular interaction between HUWE1 and BRCA1 has been further established in multiple cell types. In HEK293T cells, HUWE1 overexpression accelerated BRCA1 degradation while HUWE1 inhibition stabilized BRCA1 [29,86]. 

In response to genotoxins, an inconsistent relationship between HUWE1 and BRCA1 has been observed [29,82]. HUWE1 deficiency resulted in decreased levels of BRCA1 upon doxorubicin and y-irradiation treatment in MEFs and B-cells. These effects were attributed to insufficient levels of activated ATM secondary to HUWE1 deficiency [82]. Contrastingly, HUWE1 deficient and BRCA1-overexpressing breast epithelial cells (MCF10F) were resistant to irradiation and mitomycin c (MMC). This observation was consistent with the hypothesized role for HUWE1 in repressing BRCA1-mediated DSB repair [29]. HUWE1 deficiency thus potentially enhances BRCA1 and subsequent HR upon genotoxic stimuli. However, these data should be interpreted carefully given the context-specific variation in the relationship between HUWE1 and BRCA1.

The efficacy of genotoxins is intimately related to the cellular ability to repair DSBs. Specifically, cells that suffer from impaired HR are hypersensitive to platins and various other cancer drugs [87]. Events that influence HR do not solely affect the repair of DSBs. A growing body of evidence namely links HR to DSB-unrelated functions including RF stabilization and restart of stalled RFs, which both are of significance with regard to sensitivity to platinum-based therapy as previously discussed [57].

The direct relationship between HUWE1, BRCA1, and sensitivity to platins remains to be established. However, similarly to platins, the cytotoxic mechanism of MMC relies on DNA crosslinking. While the observed resistant phenotype upon HUWE1 KD was attributed to BRCA1 enhancement, these data should be interpreted carefully since other HUWE1 targets were not studied and no mechanistic experiments on the role of BRCA1 were conducted [29]. It is thus likely that other HUWE1 targets are at least partially responsible for the reported resistance to MMC and irradiation. Regardless of the underlying mechanism, these observations further promote HUWE1 as a sensitizer to genotoxins.

The relationship between BRCA1 and sensitivity to platins has been studied extensively in oncology. Tumors with mutant forms of BRCA1 are especially sensitive to platins [88]. In line with these reports, cisplatin resistance in breast and ovarian cancer cells has been attributed to BRCA1 wild-type (WT) overexpression [89,90]. Next to its role in HR, BRCA1 modulates the cell cycle at multiple levels. Interestingly, it promotes transcription of p21/Waf1 and p27Kip1 which can block G1/S phase progression independent of p53 [91,92]. As platins are primarily cytotoxic within S phase, BRCA1 may thus indirectly prevent transition to S phase and induce therapy resistance in this way [93]. Additionally, BRCA1 stimulates NER, which is essential in the processing of platinum-DNA adducts prior to RF collision, as discussed earlier (Figure 1) [94]. 

Whether ATM activation relies on HUWE1 in response to platins has yet to be investigated. Moreover, the exact mechanism of HUWE1-mediated ATM activation remains elusive. HUWE1 may prime ATM with specific Ub chains that are used by other factors to further phosphorylate and activate ATM. Proposing HUWE1 as an ATM activator and thereby DSB repair enhancer conflicts with the earlier suggested role for HUWE1 as a sensitizer to platins. However, ATM has pleiotropic cellular functions and its modulatory role on p53 within intrinsic apoptosis might be dominant regarding sensitivity to platins (Section 3) [33]. Additionally, impaired ATM kinase activity on targets such as BRCA1 due to HUWE1 depletion can be compensated for by ATR [95]. 

Similar to the cisplatin bladder cancer screen, a recent genome-wide CRISPR/Cas9 KO screen has evaluated regulators of the response to poly-ADP-ribose polymerase (PARP) inhibitors (PARPi) [18,96]. This type of drug has proven its effect in tumors that lack effective HR [97]. Strikingly, HUWE1 KO was among the screen’s top hits and strongly correlated to PARPi resistance in HeLa BRCA2 KO cells [96]. PARP is intimately related to DNA damage repair and functions as a mediator of various pathways. These include the repair of ssDNA damage via NER and the initiation of HR-mediated DSB repair via recruitment of proteins including BRCA1. Furthermore, PARP stabilizes the RF in the situation of replication stress [98]. HUWE1 KO was found to partially rescue defective HR in HeLa BRCA2 KO cells via overexpression of RAD51, a protein that is known for its role in DSB repair [96]. Although these data cover a different therapeutical domain, platins and PARPi rely on overlapping mechanism to trigger a cytotoxic response. Therefore, the reported relationship between HUWE1, HR, and PARPi further supports our current mechanistic view on how HUWE1 can modulate sensitivity to platins.

HUWE1 interferes with mediators of DSB repair and HR specifically. Although the reported relationship between HUWE1 and BRCA1 in response to genotoxic stressors is inconsistent, loss of HUWE1 can upregulate BRCA1. As BRCA1 is a well-known and potent inducer of cisplatin resistance, the interaction with HUWE1 may provide novel leads to enhance therapy sensitivity. However, the potential use of HUWE1 as a biomarker to assess the responsiveness to platins with regard to DSB repair requires more research. 

## 3. HUWE1 Modulates the Intrinsic Apoptotic Pathway

A severely threatened genome shifts the DDR from a reparative state towards controlled cell death [9]. Apoptotic signaling follows interconnected patterns that ultimately converge on caspase-executioners for cellular decay. Genotoxins including platins rely on the intrinsic, mitochondrial, pathway to induce cell death. Intrinsic apoptosis is regulated by a delicate balance between pro- and anti-apoptotic members of the Bcl-2 family. These control the release of cytochrome c (cyt c) into the cytoplasm [99,100]. In response to genotoxic stress, pro-apoptotic Bcl-2 induction destabilizes the mitochondrial membrane, thereby facilitating the cytoplasmatic release of cyt c and caspase activation [100]. The tumor suppressor p53 is a principal pro-apoptotic non-Bcl-2 protein and DDR effector that promotes this process [101]. Post-translational activation of p53 in response to cellular stress triggers an apoptotic cascade [102].

**Figure 4:**
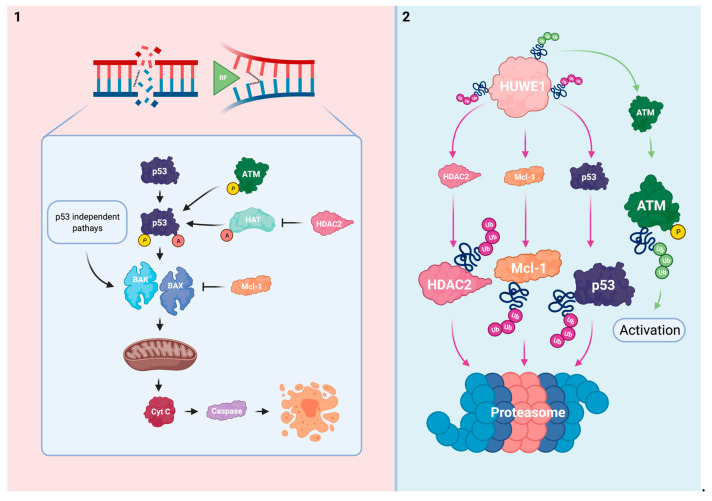
HUWE1 influences the intrinsic apoptotic pathway. (**1**) In response to irreparable DNA damage, p53 is activated via ATM-mediated phosphorylation and HAT-mediated acetylation. HDAC2 inhibits acetylation of p53. The pro-apoptotic BAK/BAX are stimulated by p53 activation and via p53 independent mechanisms. This subsequently results in mitochondrial pore formation and the cytoplasmatic release of cyt c to effectuate apoptosis. The anti-apoptotic Mcl-1 inhibits BAK/BAX. (**2**) HUWE1 negatively regulates p53, Mcl-1, and HDAC2 via polyubiquitination. HUWE1 positively regulates ATM phosphorylation via polyubiquitination. HUWE1 depletion could result in cellular immortalization and resistance to platins via target overexpression and subsequent ineffective activation of the intrinsic apoptotic pathway.

The intrinsic apoptotic pathway is influenced by the effects of HUWE1. Considering this process, HUWE1 directly regulates p53 and Mcl-1, an anti-apoptotic member of the Bcl-2 family. In addition, HUWE1 impairs the post-translational activation of p53 via regulation of ATM and histone deacetylase 2 (HDAC2) (Figure 4). HUWE1-mediated effects on the cellular ability to induce apoptosis in response to platins might be considered as a potent driver of therapy sensitivity.

P53 loss of function occurs frequently in various cancers and can lead to cellular immortalization [103]. Normally, cells maintain p53 at low levels to maintain their proliferative capabilities via mechanisms such as the mouse double minute 2 homolog (MDM2), an E3 Ub ligase. However, in response to cellular stress, p53 is rapidly phosphorylated and stabilized to block MDM2-mediated degradation [104]. In addition to phosphorylation, histone acetyl-transferase (HAT)-mediated p53 acetylation is indispensable for its activation [105]. Activated p53 stimulates multiple proteins including the Bcl-2 homologous antagonist/killer (BAK) and Bcl-2 associated X (BAX) to shift cellular balance towards a pro-apoptotic state [106,107]. However, BAK and BAX can also be activated in p53 deficient conditions due to Mcl-1 neutralization, thereby indicating the relevance of the interplay between HUWE1 and intrinsic apoptosis even in a p53 mutant tumor [108].

HUWE1 has been found to directly target p53 for proteasomal degradation in multiple models [28,30,82,109]. This reciprocal relationship at steady state seems to promote the cell’s proliferative capabilities similarly to the MDM2/p53 complex. Conversely, a positive relationship between HUWE1 and p53 has been identified in multiple thyroid cancer models. HUWE1 overexpression increased p53 stability by MDM2 downregulation in thyroid cancer (WRO) cells and mouse xenografts. Ectopic HUWE1 expression in HUWE1 KD thyroid cancer cells sensitized this model to cisplatin and other genotoxins [110]. 

ATM activation is mediated by HUWE1 in B-cells and MEFs as previously discussed. In HUWE1 deficient and p53 sufficient conditions, these cells failed to successfully trigger a p53 response cascade upon exposure to doxorubicin, etoposide, and y-irradiation due to insufficient levels of activated ATM. Interestingly, the response to dexamethasone, a p53-independent inducer of apoptosis, was not influenced by HUWE1 status in B-cells [82].

HDAC2 has been identified as a HUWE1 target for proteasomal degradation in MEFs [111]. HUWE1 deficiency impaired the ability to induce apoptosis in response to cisplatin or nutlin-3 treatment via HDAC2 enhancement in these cells. Whereas phosphorylated and acetylated p53 accumulated in HUWE1 WT-expressing controls, HUWE1 KO MEFs failed to effectuate these post-translational modifications upon genotoxic stress. Subsequently, these cells could not effectively initiate a downstream p53 response cascade. Reduction of HDAC2 to near WT levels was sufficient to normalize the stress-induced p53 cascade and re-sensitized HUWE1 KO MEFs to cisplatin and nutlin-3 treatment [111]. Taken together, these data imply that loss of HUWE1 impairs the ability to effectively trigger and effectuate p53-mediated apoptosis in response to platins and other genotoxins.

Mcl-1 exerts its anti-apoptotic function primarily by complex formation with the pro-apoptotic BAK and BAX proteins via their shared BH3 domains. Within these complexes BAK and BAX’s ability to destabilize the mitochondrial membrane is impaired [112].

HUWE1′s BH3 domain mostly resembles BAK’s BH3 domain allowing it to act as a dose-dependent regulator of Mcl-1 in HeLa cells [27,113]. Whereas the BH3 domain serves as a Mcl-1 docking station, the consecutive action of HUWE1′s catalytic HECT domain ubiquitinates Mcl-1 for proteasomal degradation [27]. Cellular Mcl-1 has been reported to accumulate in HUWE1 deficient conditions [27,111,113,114,115]. Interaction between HUWE1 and other Bcl-2 family members was not observed in HeLa cells [27,113]. However, HUWE1 KD in an ischemic cortical neuron model modulated Bcl-2 and BAX additionally to Mcl-1 which further promoted the anti-apoptotic phenotype [116]. HUWE1 might thus have a more comprehensive role in regulation of Bcl-2 members.

The direct relationship between HUWE1, Mcl-1, and the response to platins has been described in several reports [27,111,114,117]. HUWE1 KD in HeLa cells impaired the apoptotic response upon UV-irradiation, etoposide, or cisplatin exposure via Mcl-1 enhancement. Of the investigated genotoxic agents, cisplatin proved to be the least potent to trigger apoptosis [27]. In line with these findings, Mcl-1 reduction to WT levels re-sensitized HUWE1 KO MEFs to cisplatin treatment [111]. Others indicated that bile salt-induced Mcl-1 phosphorylation enhanced Mcl-1 stability by blocking HUWE1-mediated degradation in human liver cancer (HepG2) cells. The subsequent inability to effectuate apoptosis upon cisplatin treatment could be rescued by Mcl-1 KD in these cells [117]. Similarly to the observations in cell models, intestinal crypts of transgenic colorectal cancer HUWE1 KO mice displayed a decreased cisplatin sensitivity by Mcl-1 upregulation [114]. HUWE1 deficiency thus protects cells from undergoing apoptosis in response to platins and other genotoxins via Mcl-1 upregulation.

Mcl-1 is considered one of HUWE1′s principal targets in oncology. Next to HUWE1, Mcl-1 turnover is also mediated by the β-transducin repeat-containing E3 Ub protein ligase and Ub-independent pathways [118,119]. In addition to the direct evidence between the HUWE1/Mcl-1 axis and cisplatin sensitivity, Mcl-1 overexpression is a well-known inducer of resistance to platins and other genotoxins [120,121]. The potential anti-neoplastic use of Mcl-1 inhibitors is currently being investigated [122].

Non-functional mutagenic p53 can impair the apoptotic response and is linked to platinum-based therapy resistance [103,123]. However, the exact role of p53 in modulating sensitivity to platins is complex and cell context-dependent [124]. Mutagenic loss of p53 function is accompanied by conformational changes that influence its susceptibility to post-translational modifications for protein activation. Interestingly, the impaired ability to acetylate mutated p53 is linked to its decreased functionality [125]. In addition, overexpression and cytoplasmatic mislocalization of p53 have been linked to platinum-based therapy resistance by inhibiting caspase effectors [126]. Similarly to mutagenic p53, oncogenic loss of HUWE1 might decrease the cellular responsiveness to platins via impaired p53 activation. However, the reported relationship between HUWE1 and p53 varies among cell types and depends on the interplay with MDM2 [28,30,82,109,110]. This strengthens the concept that Ub network organization varies in spatiotemporal dimensions [127]. Whereas inhibition of MDM2 with nutlin-3 sensitizes cells to platins, this mechanism of action relies on enhancing p53 levels rather than promoting its post-translational activation [128,129]. Nonetheless, it highlights the potential of targeting UPS mediators to promote p53-mediated apoptosis. Complementary to its principal role in apoptotic signaling, one should note that p53 has a broad spectrum of other effects within the DDR [130].

Next to the direct evidence on HDAC2 as a cisplatin desensitizer upon loss of HUWE1, HDAC2-induced resistance to cisplatin and other DNA damaging agents is supported within other conditions [131,132]. Other HDACs are however involved in the post-translational modification of p53 which further indicates the potential existence of cellular rescue and compensation mechanisms [133]. In addition to its non-histone effects on p53, HDAC2-mediated deacetylation of histones results in DNA condensation [134]. This impairs the DNA accessibility for platins to induce DNA damage [135]. The use of HDAC inhibitors is an emerging field of cancer drug research that could be especially promising when applied synergistically with established cancer drugs such as platins [136,137]. 

Platinum’s primary mode of action is inseparably connected to the intrinsic apoptotic pathway. Altogether, the direct and indirect regulatory effects of HUWE1 on Mcl-1 and p53 propose an important role for HUWE1 in successfully triggering intrinsic apoptosis in response to platins. This suggests that HUWE1 has the potential to act as a biomarker to assess an individual’s response to platins. Moreover, stimulation of HUWE1 could induce the intrinsic apoptotic pathway which further promotes HUWE1 as a sensitizer of platinum-based therapy.

## 4. Concluding Remarks

In this review, HUWE1 has been proposed as a modulator of the sensitivity to platinum-based chemotherapy by interfering with multiple aspects of the DDR. Firstly, we discussed how HUWE1 might be involved in the processing of platinum-DNA adducts prior to RF encounter (Section 2.1). Secondly, we described how HUWE1 modulates the prominent ATR-Chk1 pathway that plays an important role in mitigating replication stress upon collision between RFs and platinum-DNA adducts (Section 2.2). Thirdly, HUWE1 has been linked to HR-mediated DSB repair that becomes active tot repair DNA damage as a consequence platinum-DNA adducts (Section 2.3). Finally, we indicated how HUWE1 activates the intrinsic apoptotic pathway in response to platins (Section 3). Taken together, these data support that HUWE1 promotes a cytotoxic response to platins and subsequently functions as a pro-apoptotic mediator to effectuate apoptosis and protect from mutagenesis. Thereby, this review provides a mechanistic framework in which HUWE1 deficiency is proposed as a potent multifactorial driver of resistance to platins. 

As ‘Evading Apoptosis’ is as a fundamental hallmark of cancer and the mechanistic link between HUWE1 and apoptosis is dominant, we hypothesize that the relationship between HUWE1 and intrinsic apoptosis is rate limiting in determining the cellular responsiveness to platins. Considering this hypothesis, even cells that do acquire significant levels of DNA damage would remain viable in HUWE1 deficient conditions due to a dysfunctional apoptotic apparatus. Although hyperactive DNA damage repair and tolerance might alter the apoptotic threshold, it is unlikely that all platinum-based therapy induced DNA damage will be correctly resolved. Additionally, DNA damage repair and tolerance mechanisms such as TLS are mutagenic and can induce further genomic instability [138]. Combined, these characteristics could facilitate the selection and expansion of aggressive tumor subclones in HUWE1 deficient conditions, thereby further driving tumor malignancy.

One of the major challenges in establishing HUWE1 as a biomarker for therapy response assessment is that its targets are also influenced by other E3 Ub ligases. Even Mcl-1, a target that shares a conserved BH3 domain with HUWE1 for docking is not solely targeted for proteasomal degradation by HUWE1. As mentioned earlier, this increases the probability that the significance of HUWE1 on protein turnover varies in spatiotemporal dimensions and HUWE1 loss of function might be compensated for by upregulation of other E3 Ub ligases. Another challenging feature in HUWE1 research is that it facilitates multiple types of ubiquitination with distinct cellular functions. To be able to use HUWE1 as a future cancer biomarker, it is therefore of essence to gain a deeper understanding on its multifaceted role across different tumor subtypes and stages of disease progression. 

Research on HUWE1′s function has primarily focused on the role of its catalytic HECT domain. However, HUWE1 contains other structural elements that are at least partially responsible for its biological behavior. Whereas the exact role of multiple domains remains elusive, the UBA domain might provide a target to stimulate HUWE1. This domain has namely been reported to enhance protein stability by preventing proteasomal degradation of Rad23 and Dsk2, both mediators of the UPS [139]. Whether HUWE1′s UBA domain exerts similar effects remains elusive. However, by mechanistically proposing HUWE1 as a sensitizer to platins, it may provide an interesting lead for future research. Another future direction could be altering HUWE1′s catalytic activity. Binding of HUWE1 with the p14ARF tumor suppressor has namely been reported to shift this conformational equilibrium toward the inactive state [140]. Whether this interaction can be blocked to promote HUWE1′s catalytic activity, thereby sensitizing to platins, has yet to be researched. However, broadly targeting of p14ARF would not be a rational strategy, since this protein is also considered to be a sensitizer to platins [141].

Cisplatin remains one of the most used cancer drugs to date. Although great advances regarding the understanding of platinum-based therapy sensitivity have been made over the past few decades, resistance remains the major hurdle in its efficacy. Therefore, it is critical to continue to broaden the knowledge on the underlying causes of resistance to platins. Moreover, the clinical quest to stratify patients prior to treatment emphasizes the urgency to discover reliable biomarkers to predict and assess individual responses. Integrating this mechanistic framework on HUWE1 as a sensitizer to platins with the recently discovered role of HUWE1 on cisplatin sensitivity in bladder cancer, support the high potential of HUWE1 to be used for such purposes. Future research on the relationship between HUWE1 and platins could generate new mechanistic insights in resistance to platins. Ultimately, HUWE1 might serve as a clinical biomarker to tailor cancer treatment strategies or for the development of new drugs, thereby improving cancer care and patient outcomes.

## Data Availability

Not applicable.
